# Piloting a brief digital behavioral intervention for adolescent sleep

**DOI:** 10.1186/s41606-025-00157-4

**Published:** 2025-10-31

**Authors:** Deniz Keskinel, Maira Karan, Katherine A. Kaplan, Riya Mirchandaney, Lauren Asarnow, Jamie M. Zeitzer

**Affiliations:** 1 Department of Psychiatry and Behavioral Sciences, Stanford University, 3801 Miranda Avenue (151Y), Palo Alto, Stanford, CA 94305 USA; 2 Department of Psychiatry and Behavioral Sciences, University of California San Francisco, San Francisco, CA USA; 3 Present Address: Department of Psychology, University of Pittsburgh, Pittsburgh, PA USA; 4 Present Address: Department of Clinical Neurology and Pediatrics, Keck School of Medicine of USC, Los Angeles, CA USA

**Keywords:** Adolescents, Sleep, Digital intervention, Mood

## Abstract

**Background:**

Adolescents worldwide suffer from inadequate sleep duration. Much of the sleep curtailment is secondary to going to sleep late and awakening early to get to school. While several interventions to shift bedtimes to an early hour have been proposed, many are not readily disseminable. The purpose of this study was to pilot the application of a digital behavioral intervention on adolescent sleep timing and subsequent effects on mood.

**Methods:**

Over a 10-week period, we studied fourteen healthy adolescents (M age = 16.1 years, SD = 1.4, 50% female) who wanted to go to sleep at an earlier hour. After a two-week baseline, participants were randomly assigned to an eight-week active or placebo light therapy condition. During the first four weeks of intervention, both groups additionally received the digital behavioral intervention, which included elements of Cognitive Behavioral Therapy for Insomnia (CBT-I), values-based clarification, and motivational interviewing. The intervention consisted of 19 short core videos and four optional values-based videos.

**Results:**

Sleep logs showed that post-intervention, as compared to baseline, both groups went to sleep earlier (CBT + active light: 7.70 ± 45.8 min; CBT + sham light: 24.8 ± 39.1 min) and slept longer (CBT + active light: 13.0 ± 47.3 min; CBT + sham light: 47.8 ± 74.6 min). Sleep latency decreased slightly in the CBT + active light (3.47 ± 9.07 min) and increased in the CBT + sham light (8.78 ± 18.2 min). Self-reported sleep quality supported these findings. No changes were observed in depressive symptoms or emotion regulation.

**Conclusions:**

Preliminary findings suggest that the current digital behavioral intervention improves self-reported measures of sleep. Further research with a larger sample size is needed to fully assess the intervention’s efficacy and long-term effects.

**Trial registration:**

Clinical Trials.gov #NCT05808179.

## Background

Compared to other stages of development, adolescence is marked by poorer and shorter sleep. As youth enter puberty, biological changes in both circadian and homeostatic processes contribute to a preference amongst adolescents for later bedand wake times (Baum et al. 2014). This delay in sleep timing conflicts with the early start times of many high schools, resulting in adolescents who go to sleep late and wake up early on school days (Blake et al. 2016). The age-related delay in sleep timing is further strengthened by social and academic pressures (Blake et al. 2017a). Adolescents often have extracurricular activities or jobs after school, stay up late to finish homework and socialize, all of which, coupled with the use of electronics (Blake et al. 2017b), further delay the timing of their sleep. Thus, psychosocial and societal factors reinforce the tendency towards circadian delay, leading to a vicious cycle (Blake et al. 2019).

In the United States, insufficient sleep among adolescents is widespread (Blom et al. 2015; Bonnar et al. 2015). Insufficient sleep has a significant impact on adolescents’ cognitive performance, physical health, and mental well-being (Cain et al. 2011). The effects of sleep loss on mental well-being may be especially impactful. Greater sleep disturbance and poorer subjective sleep is strongly associated with more depressive symptoms among adolescents (Carmona et al. 2021; Carney et al. 2012). Shortened sleep is also associated with worsened mood and difficulty regulating negative emotions in adolescents (Carskadon 2011; Carskadon et al. 2004). Taken together, to improve adolescent wellbeing across multiple domains, interventions designed to extend adolescent sleep duration require a comprehensive approach that considers both biological and behavioral factors to break the cycle of insufficient sleep.

Several interventions have been developed to improve adolescent sleep. School-based educational interventions, typically delivered by teachers and focused on psychoeducation, are among the earliest approaches. Other school-based interventions based on the motivational interviewing framework have also been developed (Chan et al. 2025). However, educational interventions have yielded mixed results, often improving students’ knowledge about sleep but failing to lead to meaningful behavioral changes (Cleary 2025). More active interventions have also been developed, such as sleep extension, cognitive behavioral therapy for insomnia (CBT-I), motivational techniques, and chronotherapy. Sleep extension interventions have demonstrated efficacy in increasing sleep duration in adolescents (Cliffe et al. 2020; Crowley and Eastman 2017). CBT-I has typically been the frontline treatment for many adult sleep issues, but the scientific literature in the adolescent population has been more limited (Cleary 2025). It involves several components, including sleep restriction, stimulus control, cognitive therapy, sleep hygiene education, and relaxation techniques. There have been a few studies that show CBT-I has been effective in improving adolescents’ sleep onset latency, sleep efficiency, and sleep duration (Crowley et al. 2018; de Bruin et al. 2015). To enhance treatment outcomes, some interventions have combined motivational interviewing with CBT-I to address the lack of motivation and create lasting change, leading to better improvements in daytime sleepiness and sleep duration than stand-alone treatments (de Bruin et al. 2018). Cognitive behavioral and mindfulness-based interventions have also proven to be effective in decreasing sleep latency in adolescents (Dewald-Kaufmann et al. 2013). Recently, hybrid treatments have emerged that integrate several different therapeutic techniques, making them more effective. The Transdiagnostic Intervention for Sleep and Circadian Dysfunction (TranS-C), which combines CBT-I, interpersonal and social rhythm therapy, motivational interviewing, and chronotherapy, has recently been developed to address a range of sleep difficulties across various at-risk populations. In adolescents with evening chronotype (tendency towards eveningness), use of TranS-C did not change total sleep time but was associated with reduced evening preference and better self-reported sleep (Van Dyk et al. 2017). Other interventions have further adapted elements of sleep optimization to include motivational techniques to encourage earlier bedtimes in adolescents (Chan et al. 2025; Garney et al. 2021). Still others have included morning light therapy to move the circadian system to an earlier hour (Goldstone et al. 2020; Gradisar et al. 2011). While approaches such as CBT-I and TranS-C show promise in improving adolescent sleep, most intervention options are administered in-person by licensed clinicians, and the supply of clinicians trained in behavioral sleep approaches is far short of demand. Further barriers to clinician-delivered sleep therapy include long waitlists, high costs, lack of insurance coverage, limited scheduling availability due to school and extracurricular activities, and dependence on adults for transportation (Gradisar et al. 2022; Gratz and Roemer 2004; Hartstein et al. 2024; Harvey et al. 2018). These challenges highlight the need for researchers to develop intervention efforts that are accessible digitally so that youth are not limited by the scarcity of clinicians or several barriers to treatment access.

There have been a limited number of studies supporting the use of digital interventions for sleep issues in adolescents (Cleary 2025; Hoyt et al. 2018; Kaplan 2019; LeBourgeois et al. 2005). Most of these studies supplement the digital intervention with weekly calls and none have specifically targeted the circadian system. Further, as expected, these interventions have been designed and applied to an adolescent population with insomnia. Several studies have examined internet delivered CBT-I interventions that incorporate active therapist support, which were found to be effective in reducing insomnia symptoms in adolescents (Ma et al. 2018; Mathews et al. 2023; Moghadam et al. 2024; O'Callaghan et al. 2021). These studies primarily involved adolescents diagnosed with insomnia, and two also included adolescents presenting with mental health problems. When given the opportunity to choose, many adolescents in fact prefer digital interventions over in-person treatment (Cleary 2025; Moghadam et al. 2024; Ogeil et al. 2020). However, these digital interventions included therapist support via weekly calls, personalized advice and feedback, and regular check-ins, which may limit their scalability and cost-effectiveness. Furthermore, many adolescents do not have insomnia yet they get an insufficient amount of sleep because of their late bedtimes coupled to enforced early wake times.

In an effort to target adolescent insufficient sleep, Kaplan et al. (2019) designed an in-person behavioral intervention combining CBT-I techniques with motivational interviewing and elements of Acceptance and Commitment Therapy (i.e., discussion on how sleep was consistent with participant-reported values). This was delivered in weekly one-on-one sessions over four weeks. When delivered alone, this behavioral therapy was able to increase total sleep time by 11 min; combined with a passive light flash therapy during sleep, meant to move the circadian system to an earlier time, this intervention increased total sleep time by 45 min per night (Paavonen et al. 2016). While this dual therapy was associated with robust increases in adolescent sleep duration, there is a need for a more accessible alternative to the often costly and time-intensive services of one-on-one therapy. Hence, expanding on the dual therapy offered in the Kaplan et al. (2019) trial, we sought to translate this behavioral therapy into a series of videos, creating an easily disseminable and accessible alternative to in-person sessions. The current study examined the combined effect of light flash therapy during sleep with an online behavioral intervention on adolescent sleep across eight weeks. We also examined the effectiveness and acceptability of the online intervention.

## Methods

### Participants

**Fig. 1:**
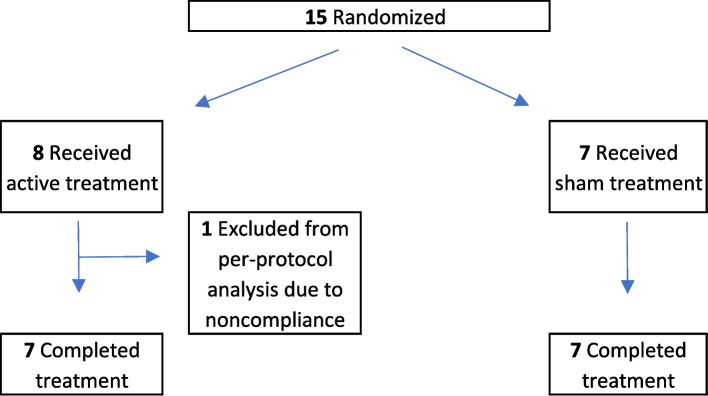
CONSORT diagram delineating the recruitment process

The current study examined 14 adolescents (M_years_ = 16.1 years, SD = 1.4; 50% female) (Table 1, Fig. 1) who were recruited by contacting school counselors, principals, teachers, and Parent-Teacher Associations who shared study flyers in e-newsletters and in classrooms. Research talks were also given by the study team at local high schools in Northern California. Recruitment took place from September 1, 2022, to February 28, 2023.

**Table 1:** Sample demographic information by intervention group

	Active	Placebo
Characteristic	(*n* = 7)	(*n* = 7)
Age, M (SD) [years]	16.4 (1.5)	15.9 (1.2)
Sex, n (%)		
Male	4 (57.1)	3 (42.9)
Female	3 (42.9)	4 (57.1)
Ethnicity, n (%)		
Not Hispanic or Latino	6 (85.7)	6 (85.7)
Hispanic or Latino	0	1 (14.3)
Unknown/do not wish to answer	1 (14.3)	0
Race, n (%)		
White	4 (57.1)	3 (42.9)
More than one race	1 (14.3)	3 (42.9)
Asian	1 (14.3)	1 (14.3)
Unknown/do not wish to answer	1 (14.3)	0

The target sample of participants was defined as adolescents aged 14–18 years old who were enrolled full-time in high school and had both the desire and ability to go to bed one hour earlier (i.e., no structural barriers prevented them from doing so). Exclusion criteria entailed active use of any medication prescribed for sleep (including melatonin), currently receiving a behavioral treatment for sleep, and sleeping only in a prone (face-down) position. Interested and eligible adolescents completed an assent form and informed consent was obtained from their parent or guardian. All study procedures were approved by Stanford University’s Institutional Review Board.

### Procedure

**Fig. 2:**
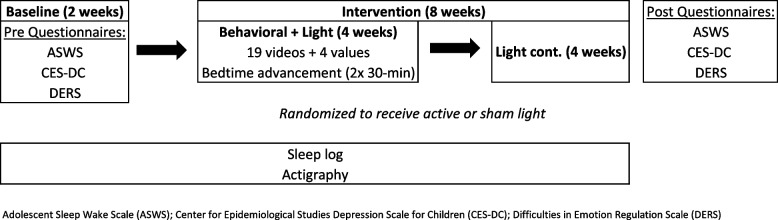
Diagram of the study protocol

The study lasted for 10 weeks: two weeks of baseline measurement followed by four weeks of combined behavioral and light treatment, then four weeks of only light treatment (Fig. 2). Data were collected during the academic school year between January and May 2023. Participants were assigned to one of two groups: (1) active light + behavior therapy or (2) placebo light + behavior therapy. Participants were randomly assigned using a double-blind procedure. Both groups received the same online behavioral intervention for the first four weeks of the intervention and differed only in that one received an active light intervention and the other received a placebo light intervention (see below).

During the two-week baseline, participants completed electronic questionnaires and daily morning sleep logs (Consensus Sleep Diary (Paruthi et al. 2016)). Following baseline, participants began an eight-week intervention. In the first four weeks, participants received both the behavioral and light interventions. During the latter four weeks, participants only received the light intervention. During the eight-week intervention, participants complete daily morning sleep logs and wore an actigraph at night (ActLumus, Condor Instruments, São Paulo Brazil). A lab technician blind to the intervention group allocation visited each participant’s home to install the intervention lamps (see below) in bedrooms and deliver the actigraph. Participants received automated (REDCap via Twilio) reminders of their bedtime across four weeks of the intervention via short message service (SMS) 90 min before their bedtime, the timing of which was gradually advanced during the intervention. Since the study was completed during the academic year, participants’ holidays, vacations, and days away from the home were documented. At the end of the intervention, participants were asked to complete the same questionnaires as were administered during baseline. All self-reported data (i.e., questionnaires, sleep diaries) were collected and managed electronically via REDCap tools hosted at Stanford University.

### Behavioral intervention

**Fig. 3:**
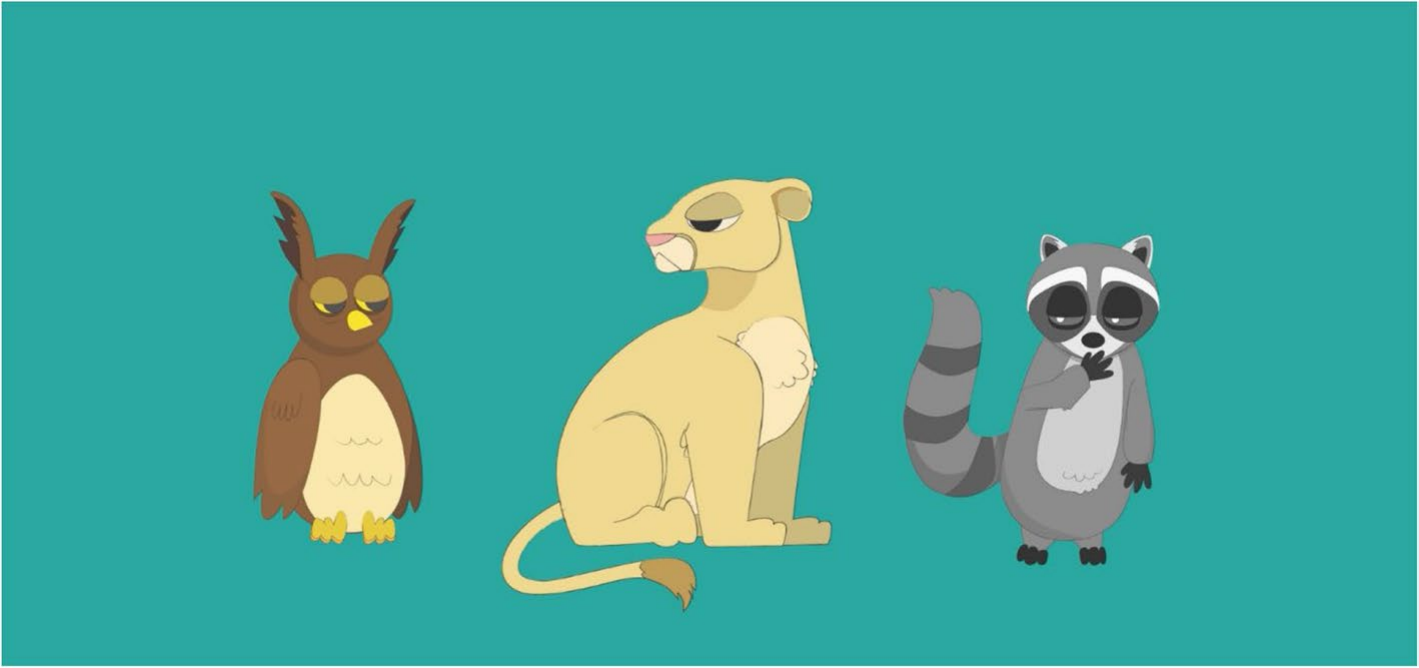
Three archetypal characters are used in the video to represent different sleep problems. The late night owl (left), the anxious mountain lion (middle), and the gamer raccoon (right)

Authors (L.A., K.K., D.K., R.M.) converted the in-person content of the previously described (Paavonen et al. 2016) one-on-one behavior sessions into a series of short, animated videos. The behavioral intervention had four components: 1) education on the circadian system, the impact of light, and physiological processes; 2) the role of sleep in domains relevant to adolescents (e.g., academics, physical health, mood, hobbies); 3) sleep hygiene and stimulus control, well-established insomnia treatment components designed to improve sleep and associated contextual cues; and 4) activity scheduling to wake up earlier on weekends and anticipate obstacles. Motivational interviewing and values-based clarification were central features of the intervention. There were 19 videos in total; a link to one video was sent out at 5 pm, every weekday, Monday through Friday, over the course of four weeks via text. Participants could choose when to watch the videos, the timing of which was automatically tracked in REDCap. The videos were on average M (SD) = 2:25 (0:28) minutes in duration. There were three archetypal characters in the videos, each with different presenting sleep problems and personalities: Livia the Lion (a straight-A student with many extracurriculars whose anxiety keeps her up at night), Otis the Owl (a night owl who enjoys staying up late), and Ryan the Raccoon (a gamer who has poor sleep hygiene) (Fig.3). The goal was to have the participants identify with one of the characters that they felt were similar to themselves in qualities and presenting sleep issues. Each video was followed by 2–4 questions for the participant to answer, as both a means of personalization and a manipulation check to ensure understanding. During baseline, participants were introduced to the idea of values and asked to list which of the following life domains mattered to them: 1) academics; 2) physical health; 3) mood; and 4) hobbies. Based on their responses, voice-over digital presentations were sent out that focused on the role of sleep in these domains. To increase the motivation of participants to start making changes to their sleep, these values-based videos were sent out during the first week of the intervention. During baseline, participants were also asked about additional factors that could be influencing their sleep, including sharing a room/noise, late night sports practices, variable wake times, awakenings at night, sleep safety concerns, and alcohol/cannabis use. Based on their answers, six additional worksheets were sent out during the last week of the intervention that discussed strategies for dealing with these specific sleep issues. Lastly, one video was sent out to parents to help them understand and guide them on how to support their teen during the behavioral intervention.

Initial bedtime was calculated as the average weekday bedtime during the two-week baseline. During Week 1 of the behavioral intervention, bedtimes were kept the same. During Weeks 2 and 3, bedtimes were advanced by 30 min. During Week 4, they were given the option to either advance their bedtime by another 30 min or to keep it as is. At the beginning of each week, the participants were informed of their new target bedtime and asked to communicate if they did not agree with the suggested bedtime. Every evening during the behavioral intervention, SMS reminders were sent out 1.5 h before their target bedtime.

### Light intervention

The light intervention was identical to that used in our previous intervention study (Paavonen et al. 2016). Participants were randomly assigned to the active or placebo light condition for the entirety of the eight-week intervention. In the active condition, daily light therapy was administered through a sequence of brief light flashes timed to begin an hour before participants' habitual wake time, as measured at baseline. Light at this time is expected to advance the phase (time) of the circadian clock (Roach et al. 2021), which would enable earlier bedtimes by shifting the peak circadian alertness signal to an earlier hour (Short and Chee 2019). The two-millisecond light flashes were administered every 20 s through a customized xenon lamp (× 500, Moflash Signalling, Birmingham UK), with the intensity of each flash being ~ 4000 lx of broad-spectrum white light (approximately 200–600 lx reaching the cornea after filtration by the closed eyelid). In the placebo condition, participants’ lamps were programmed to flash for only one minute (i.e., three flashes) one hour before habitual wake time, a stimulus insufficient to shift the circadian system. The lamps were connected to timers that were scheduled to deliver either the active (60 min) or placebo (1 min) light flash sequence; timers were programmed using baseline sleep data by an independent researcher who was not involved in the study in any other capacity. Lamps were placed in each participant’s bedroom as close as possible to their place of sleep and this distance was measured for each participant during a house visit (*M* = 1.76 m, *SD* = 2.46 m).

### Measures

During baseline and the last week of intervention, overall sleep quality, depressive symptomatology, and emotion regulation were captured through validated questionnaires. Subjective sleep quality was determined with the Adolescent Sleep Wake Scale (ASWS) (Short et al. 2020), which yields a total score and scores on five subscales: going to bed, falling asleep, maintaining sleep, reinitiating sleep, and returning to wakefulness. Higher scores indicate worse sleep quality. Self-reported depressive symptoms were assessed using the Center for Epidemiological Studies Depression Scale for Children (CESD) (Strogatz et al. 1987), with higher scores indicating increased symptoms of depression. Emotion regulation problems were examined with the Difficulties in Emotion Regulation Scale (DERS) (Vargas et al. 2023), with higher scores indicating greater problems with emotion regulation.

Self-reporting of nightly sleep characteristics (time initiating and completing sleep, sleep onset latency) was reported each morning through an SMS text reminder system. Objective sleep was recorded using actigraph watches (ActLumus, Condor Instruments, São Paulo, Brazil) that measure arm movement that can be imputed as wake or sleep in 60-s epochs (ActStudio, Condor Instruments). All valid data were used in the scoring process, with the exceptions of individual nights on which there was a major mismatch between the subjective sleep log and the objective actigraph data, or if there were no sleep log data present. Also excluded were the transition between daylight savings plus the next two nights and overseas travel plus five additional nights. School days, free days, holidays, and vacations were denoted.

At baseline, all participants completed demographic questionnaires. During the last week of treatment, all participants were offered the opportunity to complete an exit interview.

### Statistical analysis

To account for daily repeated measures of sleep across 10 weeks, sleep data were analyzed through a multilevel modeling framework. Days (level 1) were nested within participants (level 2) to estimate the effect of the intervention on sleep outcome measures across 10 weeks. We specified random intercepts in the multilevel models to account for individual differences in baseline sleep. Specifically, we modeled daily levels of change in sleep (from averaged baseline values) as a function of intervention group, day, and a random intercept for each participant. Models accounted for demographic covariates such as school grade, sex, race, and parent education. Self-report questionnaire data from pre- and post-intervention were analyzed using linear mixed models. All variables that were examined met assumptions of normality. Effect sizes were calculated using Cohen’s *d* for statistically significant results. When multiple comparisons were made within the same measure (i.e., ASWS), the significance value for an individual test was adjusted using the Benjamini–Hochberg procedure. All sleep outcomes were assessed for weekend versus weekday differences; however, no statistically significant differences emerged, thus this variable was excluded from reported analyses. Data are presented as mean ± standard deviation (SD) unless otherwise noted.

All procedures were overseen by the Stanford University Institutional Review Board and comported to the principles laid out in the Declaration of Helsinki. This study is a pilot of the full study posted on clinicaltrials.gov (NCT05808179).

## Results

### Videos

Of the 14 participants, twelve completed all 19 videos, one completed 16 of the 19 videos, and one completed 17 of the 19 videos. Most participants, however, did not watch the videos on the day they were sent, rather they watched the videos 4.87 ± 3.38 days after they were sent.

### Sleep diaries

**Fig. 4:**
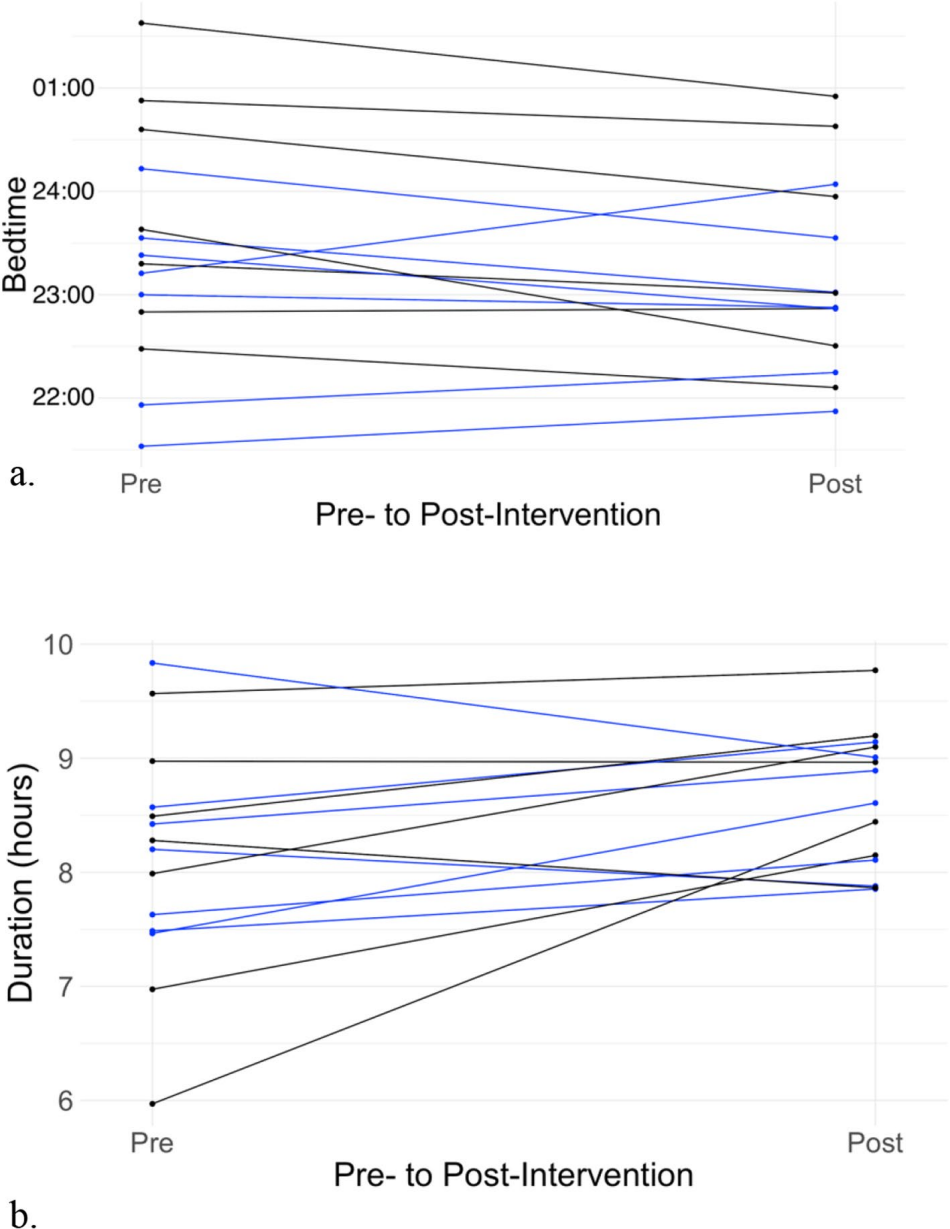
Individual trajectories in diary-based (**a**) bedtime and (**b**) sleep duration pre- versus post-intervention. Individuals in the active flash (blue) and placebo flash (black) condition are indicated

**Fig. 5:**
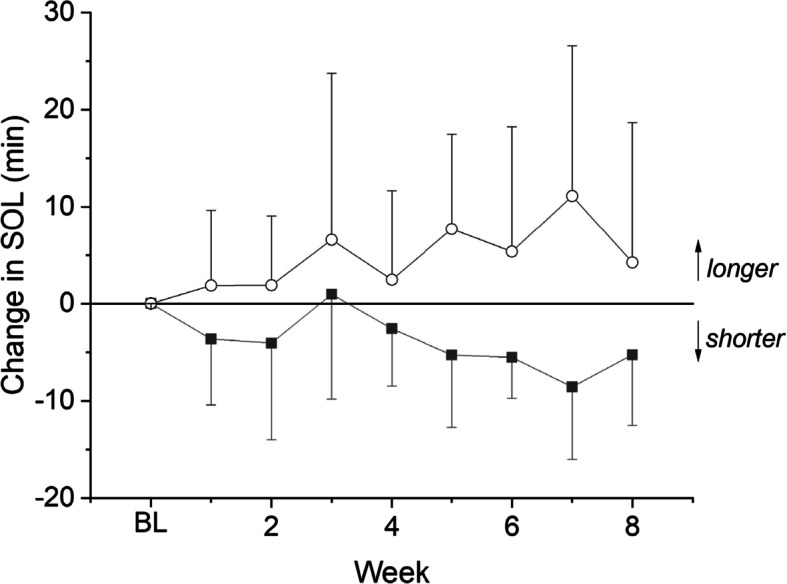
Change in sleep onset latency from baseline (BL) across eight treatment weeks in participants exposed to CBT + active light (closed box) and CBT + sham light (open circle)

The teens in this pilot study were recruited for their interest in going to sleep at an earlier hour to obtain more sleep. Per sleep diaries, the adolescents generally slept around 8 h per night and had a nearly 30-min sleep onset latency (Table 2). Bedtimes were typically in the 11 pm-midnight range (Table 2). Multilevel models did not indicate statistically significant interactions between light therapy condition and bedtime (*b* = 0.47, SE = 0.25, *p* = 0.084), waketime (*b* = −0.47, SE = 0.43, *p* = 0.299), or sleep latency (*b* = −10.17, SE = 5.84, *p* = 0.107). While there was considerable variability, according to sleep logs, as compared to individuals’ baselines (Table 2, Fig. 4), participants in both groups went to sleep earlier (CBT + active light: 7.70 ± 45.8 min; CBT + sham light: 24.8 ± 39.1 min), got up later (CBT + active light: 5.31 ± 28.1 min; CBT + sham light: 14.4 ± 74.9 min), and achieved more nightly sleep (CBT + active light: 13.0 ± 47.3 min; CBT + sham light: 47.8 ± 74.6 min). Though no significant interaction effects were observed, sleep latency was slightly shorter in the CBT + active light (3.47 ± 9.07 min shorter) and slightly longer in the CBT + sham light (8.78 ± 18.2 min longer) (Fig. 5).

**Table 2:** Sleep by condition and time. Data are presented as mean (SD)

	CBT + active light		CBT + sham light	
	Baseline	Post-Intervention	Baseline	Post-Intervention
Bedtime, HH:MM	23:00 (0:56)	22:54 (0:44)	23:54 (1:09)	23:24 (1:05)
Sleep Duration, hr (SD)	8.23 (0.84)	8.50 (0.55)	8.03 (1.22)	8.78 (0.66)
Sleep Latency, min	28.03 (16.04)	24.92 (12.33)	30.42 (20.45)	34.45 (23.92)
Waketime, HH:MM	7:13 (0:46)	7:26 (0:37)	7:57 (0:51)	8:10 (0:54)

### Actigraphy

We could not compare the sleep log data with the actigraphy data as, due to power failures in the watches, 39.7% of the overnight sleep data were not captured.

### Questionnaires

Two of the individuals in the placebo group declined to complete post-treatment questionnaires. Scores on the ASWS were not different between the two groups (*p* = 0.81, linear mixed model) though changed over time (*p* < 0.05, linear mixed model). Data from the ASWS supported the sleep log findings, with significant improvements in the total score and all subscale scores, except ‘maintain sleep’ (shown collapsed across treatment groups in Table 3). We did not observe changes over the course of the protocol in either depressive symptoms (CESD) or emotion regulation (DERS) (Table 3).

**Table 3:** Changes in questionnaires pre- to post-intervention across all participants

	Pre-Intervention		Post-Intervention		Paired t-test		Effect size
	Mean	SD	Mean	SD	t(11)	*p*	Cohen’s *d*
ASWS Going to bed	4.07	0.90	3.03	1.01	4.36	.001^*^	1.08
ASWS Falling asleep	3.51	0.81	2.94	0.81	3.06	.011^*^	0.70
ASWS Maintaining sleep	2.51	0.87	2.32	0.90	0.93	.370	0.21
ASWS: Reinitiating sleep	2.71	0.75	2.18	0.85	4.79	.0006^*^	0.66
ASWS: Returning to wake	4.28	1.00	3.78	1.04	2.66	.022^*^	0.49
ASWS Total	3.42	0.57	2.85	0.54	5.53	.0002^*^	1.03
CESD Total	18.4	11.2	14.6	5.30	1.51	.159	0.44
DERS Total	82.7	14.6	81.5	14.2	0.298	.772	0.083

^*^To control for multiple comparisons, the Benjamini–Hochberg procedure was applied with a false discovery rate (Q) =.05

### Qualitative feedback

Of the 14 participants, 8 completed an optional exit interview. Overall, participants reported a positive experience with the intervention videos. The videos were described as educational, informative, and helpful in improving their sleep. One participant noted, “It was informative and didn't take up too much time and overall it was helpful.” Three participants appreciated the brevity of the videos, while another noted that the videos felt like they were, “aimed at middle schools [sic] and not high schoolers.” Participants shared that the videos helped them learn more about sleep, and some mentioned that they saw improvements in their sleep quality as a result. Specific positive feedback included: “I liked how they were short and sweet and easy to pay attention to.” When asked about the most useful aspects of the intervention, the participants highlighted the relatability of the characters, maintaining a sleep schedule, the recaps at the end, and the general knowledge shared in the videos. One participant stated, “I thought it was useful to find out why this stuff works and how we can be better at having sleep schedules.”

Participants were also asked to provide feedback on what they found least useful. Some participants mentioned that certain advice felt overly simplistic. For instance, one participant stated, “The information about how your body works when you get less sleep or take naps or drink caffeine was least useful,” while another said it was, “obvious information in the videos.” One noted that the backstory of the characters was not helpful, while another commented that the videos were “a little too youthful.” One participant noted, “It didn’t have enough information on how to stay motivated to get more sleep.” Regarding potential changes to the intervention, three participants stated that they would not change anything. One participant suggested incorporating check-ins with a person to stay on track with going to bed, another recommended shortening the videos even further, and one proposed adjusting the frequency of the videos. The feedback on the frequency and timing of the videos was mixed. Four participants reported that it worked well for them, while three others felt the frequency was too high. Overall, all the respondents indicated that they would recommend the intervention to others seeking to improve their sleep. They expressed that the intervention provided valuable tips and reinforced healthy sleep habits. One participant stated they would recommend it as it “helps you get to bed earlier which lets you get more sleep.”

## Discussion

The current study piloted an online behavioral sleep intervention for adolescents that would be easy to disseminate, relatable to the adolescent population, and grounded in the science of CBT-I, sleep-related values, and motivational interviewing. By extending and converting the in-person behavioral intervention protocol developed by Kaplan et al. (2019) into a series of videos, this new protocol could allow youth to gain sleep education and behavioral techniques to improve their sleep from their home. As a pilot study, we did not power the sample size to account for the significant variability in sleep data that we have observed in the past. The medium to large effect sizes we observed in our questionnaire data were also consistent with what has been found in other cognitive-behavioral interventions for sleep among adolescents (Crowley et al. 2018; Van Dyk et al. 2017; Vrabec et al. 2025; Weissman et al. 1980).

In addition to examining improvements in sleep, we also assessed associated changes in depressive symptoms and emotion regulation. While not powered to detect such changes, there was evidence to suggest a larger study might reveal significant improvements consistent with a medium effect size. Alterations in mood through modifying sleep could also take longer to instantiate necessitating longer protocols to observe such changes. Such work will also be critical to enhance our understanding of the role that sleep plays in the development of depression during adolescence (Wheaton et al. 2015).

Unlike in the Kaplan et al. study (2019), we did not see a significant difference in total sleep time in adolescents exposed to a nightly flash sequence as compared to those who were exposed to a sham sequence. We did, however, see preliminary evidence for a shortened sleep onset latency in the flash condition and a lengthened sleep latency in the placebo condition. The difference was quite small but did move in the direction predicted by the Kaplan study. Confirmation with changes in the timing of melatonin are necessary to determine whether the change in sleep latency or any change in sleep timing are associated with a shifted circadian clock. We may have been limited in our ability to observe differences between conditions as many of the participants were already sleeping eight or more hours and were looking more to optimize their sleep rather than enable substantively more sleep.

The small sample size in this study prevents us from making any definitive conclusions on the effectiveness of this therapy in changing sleep or mood in adolescents. A larger sample size would also allow us to examine the moderating effects of the timing (e.g., are those with earlier bedtimes less likely to move bedtimes earlier) and duration of sleep (e.g., are those with more sleep less likely to increase total sleep time) at baseline and degree of sleep-related impairments at baseline. While it would be valuable to directly compare this digital intervention with the original in-person intervention, a non-inferiority design would require a very large number of participants and could be important if the digital intervention were to be established as an effective adjunct to the light therapy.

## Conclusions

We believe that the pilot study data are consistent with our digital intervention potentially being effective at eliciting changes in sleep timing and possibly mood in adolescents. Whether this is effective in a larger population and can be maintained over the long term remains to be determined.

## Data Availability

The dataset supporting the conclusions of this article is available in the Dryad repository, https://doi.org/10.5061/dryad.sxksn03gb.

## Abbreviations

ASWS: Adolescent Sleep Wake Scale
CBT: Cognitive behavioral therapy
CBT-I: Cognitive behavioral therapy for insomnia
CESD: Center for Epidemiological Studies - Depression
DERS: Difficulties in Emotino Regulation Scale
SMS: Short message service
TransS-C: Transdiagnostic Intervention for Sleep and Circadian Dysfunctino
